# Community-based hearing aid fitting model for adults in low-income communities facilitated by community health workers: a feasibility study

**DOI:** 10.1080/16549716.2025.2545630

**Published:** 2025-09-05

**Authors:** Bopane Mothemela, Caitlin Frisby, Faheema Mahomed Asmail, Tersia de Kock, David R. Moore, Vinaya Manchaiah, De Wet Swanepoel

**Affiliations:** a Department of Speech-language Pathology and Audiology, University of Pretoria, Pretoria, South Africa; b Virtual Hearing Lab, Collaborative initiative between University of Colorado School of Medicine, Aurora, CO, USA; c HearX Group, Pretoria, South Africa; d Communication Sciences Research Center, Cincinnati Children’s Hospital Medical Centre, Cincinnati, USA; e Manchester Centre for Audiology and Deafness, University of Manchester, Manchester, UK; f Department of Otolaryngology–Head and Neck Surgery, University of Colorado School of Medicine, Aurora, CO, USA; g UCHealth Hearing and Balance, University of Colorado Hospital, Aurora, CO, USA; h Department of Speech and Hearing, School of Allied Health Sciences, Manipal Academy of Higher Education, Manipal, India; i Ear Science Institute Australia, Subiaco, WA, Australia

**Keywords:** Hearing loss, hearing aids, community health workers, mhealth, Low- and middle-income countries

## Abstract

**Background:**

Hearing loss affects more than 1.5 billion people worldwide, yet fewer than 10% of those who could benefit from hearing aids are able to access them. Barriers such as high costs, limited availability, and a critical shortage of trained professionals in low- and middle-income countries contribute to this gap, while emerging models of care—such as task-shifting to community healthcare workers (CHWs) supported by Health technologies—show promise in improving access, affordability, and outcomes in underserved communities.

**Objective:**

To determine the feasibility and outcomes of a community-based hearing aid fitting model designed for low-income communities facilitated by community health workers (CHWs).

**Method:**

Using Bowen’s framework, feasibility was evaluated according to service delivery and patient outcomes. A total of 25 (six male) participants were fitted with bilateral GoPrime preset, over-the-counter (OTC) hearing aids by CHWs in Khayelitsha, a low-income community in Cape Town, South Africa. Benefit and satisfaction were measured using the International Outcome Inventory forHearing Aids (IOI-HA). An open-ended survey used to obtain their perceptions on the impact of the hearing aids on communication, the mHealth program, and willingness to purchase the hearing aids was analyzed using inductive thematic analysis.

**Results:**

Pure Tone Average across participants was 57.3 dB HL (11.5 SD) in the left ear and 54 dB HL (14.2 SD) int he right ear. The majority of participants self-reported positive outcomes while using hearing aids, including good hearing in background noise. IOI-HA showed above standardized average scores of 3.91 for daily use, 4.46 for benefit, and 4.58 for satisfaction. 92% of the participants reported the hearing aids as extremely helpful, with 87.5% recommending hearing aids for others with hearing loss. Additionally, participants reported positive experiences with them Health support program and described the program as clear and helpful.

**Conclusion:**

The community-based hearing aid fitting model is feasible and demonstrated positive hearing aid outcomes in a low-income community. This approach, supported bymHealth technologies and CHWs, presents a promising solution to address the hearing care gap in low- and middle-income countries (LMICs).

## PAPER CONTEXT

- **Main findings:** This manuscript outlines the feasibility of an innovative community-based hearing aid fitting model in a low- and middle-income community setting.
- **Added knowledge:** This study is part of the field research that informed the recently released WHO Guideline on Provision of Hearing Aids in Low-income settings.
- **Global health impact for policy and action:** The model employed in this study has potential to improve access and affordability of hearing healthcare services.

## Background

Hearing loss affects more than 1.5 billion people globally [1]. Over four hundred million of these individuals could benefit from intervention with hearing aids [2]. Although shown to be effective in the treatment of hearing loss [3], hearing aids are inaccessible to most people with hearing loss [4]. Globally, only about 10% of those who could benefit from hearing aids make use of hearing aids [1,5]. Major barriers contributing to the inaccessibility of hearing healthcare emanate from a lack of trained professionals, centralized models of hearing care, and the cost of traditional clinical equipment [1,6,7]. Even if some elements of care are available (e.g. hearing assessment), hearing aids are often unaffordable [8,9]. Factors such as limited availability of hearing aid manufacturers, regulation, bureaucracy, and high-profit markups contribute to the high cost of hearing aids in LMICs (Low- and middle-income countries) [10].

Recently, different approaches to hearing healthcare have been suggested and prioritized to improve access to hearing healthcare [7,11]. These approaches include task-shifting, recommended by the World Health Organization (WHO), where trained community healthcare workers (CHWs) are utilized to address the shortage of hearing healthcare professionals [1,12–14]. Availability of audiologists varies across regions, based on income level, according to WHO (2013), with only 5.2% of low-income countries and 27.7% of LMICs reporting more than one audiologist per 1 million people. Latest evidence on the shortage of audiologists has recently been reported by Kamenov et al. [15], showing that a majority of countries in the sub-Saharan region of Africa have less than one hearing healthcare professional per million people. In contrast, most European countries have more than 50 times as many audiologists.

Employment of CHWs in the provision of hearing healthcare services, supported by mHealth technologies, promotes a variety of service delivery models that could improve access compared to traditional audiological best practice. The feasibility of these alternative service delivery models has been studied by Nieman et al. [16]. They assessed the feasibility of Hearing Health Equity through Accessible Research and Solutions (HEARS). This was a community-based intervention involving screening, provision of personal sound amplification products (PSAPs), and communication education. The HEARS intervention was accepted by the community and found to be feasible for implementation. Participants self-reported improvements in hearing and communication following intervention. In a follow-up randomized controlled study of the HEARS intervention by Nieman et al. [11], an intervention group was led by CHWs and provided with low-cost PSAPs and instructions. A waitlist control group was included, receiving the intervention only after reaching the 3-month trial endpoint. Twelve-month post-intervention data were collected for both groups, with results indicating significant improvements in self-perceived communication measured by changes in the Hearing Handicap Inventory for the Elderly – Screening Version score (HHIE-S) within the intervention group.

The feasibility of an adult community-based rehabilitation (CBR) model, providing self-programmed hearing aids, CHW support, and mHealth technologies, was evaluated in a recent study by Frisby et al. [17]. The mHealth technologies included a smartphone-based video otoscope equipped with artificial intelligence (AI) image classification and automated in-situ pure-tone audiometry (at frequencies of 0.5, 1, 2, and 4 kHz) facilitated by Lexie Lumen hearing aids (hearX Group, South Africa). The study demonstrated positive hearing aid outcomes measured through the IOI-HA. Frisby et al. [18] also evaluated the feasibility of an mHealth acclimatization and support program using qualitative open-ended measures. The participants described the program as helpful, supportive, informative, sufficient, and clear at 45 days and six months follow-ups. A recent feasibility randomized controlled trial conducted by Coco et al. [19] found improved listening self-efficacy measured through the Self Efficacy for Situational Communication Management Questionnaire in the experimental group, facilitated by CHWs, compared to the control group, facilitated by trained student facilitators. These studies demonstrate the feasibility and efficacy of CHW-based service delivery models.

Despite growth in research interest in hearing aid service delivery models focused on community-based hearing care, there are limited implementation and feasibility studies on these service delivery models within LMICs. Bowen et al. [20] proposed a rigorous framework for assessing feasibility study design, outlining key areas of focus, sample outcomes, and suggested sample designs. Following that framework, this study primarily aimed to evaluate the feasibility and outcomes of a community-based hearing aid fitting model facilitated by community health workers (CHWs) in a low-income community. This included assessing the acceptability, practicality, and effectiveness of this model in delivering hearing care and improving self-reported hearing aid outcomes. Additionally, a secondary aim was to explore the role of mHealth support in hearing aid uptake and use, user confidence, perceptions of the CHW-led fitting process, and the affordability and willingness to pay for hearing aids in a low-income setting.

## Method

### Study design

The Bowen framework provides guidance for designing feasibility studies with consideration for eight focus areas, including acceptability, demand, implementation, practicality, adaptation, integration, expansion, and limited-efficacy testing [20]. Among these, this study sought to address the components of the feasibility of the community-based hearing aid fitting model, including acceptability, demand, practicality and adaptability. Cross-sectional surveys were employed to evaluate the feasibility of the model through patient outcomes. These surveys aimed to capture the patients’ self-reported outcomes and views of the model, considering the Bowen Framework focus areas. Institutional review board approval was obtained from the University of Pretoria Humanities Ethics Board (HUM011/0822).

### Participants

Three CHWs with four years’ experience in providing hearing healthcare services in low-income communities were recruited to recruit participants and conduct hearing screenings, assessments, hearing aid fittings, and follow-up. These CHWs are employed by a South African-based Non-Government Organization (NGO), the hearX Foundation, which provides access to hearing healthcare services in low-income communities. One qualified audiologist, the program manager from the Foundation, provided supervision and guidance to the CHWs when necessary. The CHWs provided hearing healthcare services from hearing testing to hearing aid fitting and follow-ups in Khayelitsha, Western Cape, South Africa. Khayelitsha is a low-income community in Western Cape, with an estimated population of about 400,000, living in 120,000 households of which 45% are formal dwellings [21]. Participants from this community were sampled through convenience sampling, where adults (18 years and above) with self-suspected hearing loss were recruited through self-report and community referral.

### Study apparatus and materials

For audiometric testing, an automated smartphone-based audiometer, the hearTest™ (hearX Group, South Africa) application, recognized for its effectiveness in diagnostic testing [22], was used with headphones. A smartphone-based video-otoscope with AI imaging capabilities, the HearScope™ (hearX Group, South Africa) was used to examine the ear. In-the-ear OTC hearing aids (GoPrime^TM^, hearX Group, South Africa) were used to fit eligible community members. These hearing aids are rechargeable, offer six channels, 12 bands, three pre-set programs, noise reduction, feedback cancellation, a memory recall function, adjustable volume and cost less than $100. Outcome measures, including the isiXhosa translated International Outcome Inventory for Hearing Aids (IOI-HA) [17] and a four-week follow-up survey (Appendix 1), were used to evaluate participant outcomes with hearing aids. The four-week follow-up survey contained open-ended questions on the impact of the hearing aids on communication, the mHealth program, and willingness to purchase the hearing aids. The mHealth program comprises of compiled messages containing information on hearing health, device management and use which are distributed through WhatsApp and text messages.

### Study procedures

**Figure 1.**
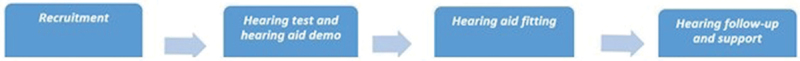
Illustration of the hearing aid service-delivery model.

The following CBR service delivery model components included (1) recruitment, (2) hearing assessment and hearing aid demonstration, (3) hearing aid fitting, and (4) follow-up and support (see Figure 1). Several recommended best practices for community-delivered hearing healthcare, as outlined by [23], were incorporated into the implementation of this study. These included the use of trained and experienced CHWs, the integration of mHealth technologies, ongoing education facilitated by the mHealth support program, and the provision of referrals to the onsite nurse for the management of ear diseases.

#### Recruitment phase

CHWs identified 188 adults with suspected self-reported hearing loss through self-report and community referrals. Written consent was obtained to conduct hearing assessments to identify and diagnose hearing loss.

#### Hearing assessment and hearing aid demonstration

Smartphone video-otoscopy was conducted by CHWs. The hearScope^TM^ was used to inspect the outer ear for disease (e.g. wax impaction, perforation, or ear infection). Images were classified by AI algorithms and uploaded to cloud storage. Where ear diseases were identified, individuals were referred to an onsite nurse.

Smartphone-based audiometry, the hearTest™ (hearX Group, South Africa), was used to evaluate hearing sensitivity in octave steps between 125 Hz and 8000 Hz. Participants were asked to raise their hand or push a button on the smartphone every time they heard a tone, even if it was soft. Participants with confirmed hearing loss (26–85 dB HL) were offered an opportunity to experience listening through the hearing aids on a program of their choice.

#### Hearing aid fitting

Twenty-five participants who met the inclusion criteria were fitted bilaterally by CHWs with hearing aids on a set program of their choice that they felt was most comfortable. Inclusion criteria included: (a) ≥18 years of age, (b) bilateral hearing loss (4FA PTA; 26–85 dB HL), (c) have access to the smartphone application WhatsApp, or SMSs (either themselves or a household member), and d) willing to be contacted for interviews. The participants were orientated regarding user-operated controls and device maintenance. The CHWs facilitated the hearing aid fittings.

#### Follow-up and support

A mHealth program was offered for 30 days to participants fitted with the hearing aids in the form of 14 WhatsApp or text messages sent on certain scheduled days (day 1, 2,4, 5, 8, 10, 12, 15, 17, 19, 22, 24, 26, 27). Information regarding hearing health, device management and use was provided in a manner accessible to the community members, such as SMS or WhatsApp messaging service. Participants received an in-person follow-up with the CHW 30 days after the hearing aid fitting. Outcome measures, including the IsiXhosa translated IOI-HA and a non-standardized hearing aid outcome survey (Appendix 1), were administered to the participants 30 weeks post-fitting.

### Data analysis

Raw data were exported to Microsoft Excel spreadsheets and the program Statistical Package for the Social Sciences [24]. Descriptive statistics, including mean and standard deviations, were determined for participant age, gender, degree of hearing loss, and the IOI-HA scores. Qualitative questions from the survey were analyzed by the first author (BM) using inductive thematic analysis to determine emerging themes. For quality control, the second author (CF) reviewed the themes, and any discrepancies were resolved through discussion.

## RESULTS

### Participant characteristics

Of the 25 participants fitted with hearing aids, 24 attended 30 days follow-up appointment. One participant withdrew from the study due to personal unforeseen circumstances. Participant demographics are shown in Table 1.

**Table 1.** Participant demographics and hearing thresholds for those fitted with hearing aids (25).

Demographic variable	Adults fitted with hearing aids (n = 25)
Age	76.5 (9.2 SD)
Gender	19 Female; 6 Male
PTA left	57.3 (11.5 SD)
PTA right	54.0 (14.2 SD)
*Degree of hearing loss*	
Mild (25–40 dB HL)	2 (8%)
Moderate (41–60 dB HL)	15 (60%)
Moderately severe (61–80 dB HL)	7 (28%)
Severe (81+ dB HL)	1 (4%)

### Self-reported hearing aid outcomes

Seventeen (70%) of the participants were wearing their hearing aids upon arrival for the follow-up visit. 25% of the participants reported wearing their hearing aids always, while 58% reported wearing them often. 92% of participants reported that they found their hearing aids extremely helpful, and 87.5% mentioned that they would ‘definitely recommend’ the hearing aids. With the IOI-HA, participants obtained a score of 3.91 for daily hearing aid use. Furthermore, the average scores for items including hearing aid benefit and satisfaction were 4.46 and 4.58, respectively. The total IOI-HA score obtained is 32.08, indicating above average hearing aid outcomes (Table 2). These results are above the statistical norms of 3.73 for hearing aid use, 3.39 for hearing aid benefit, and 3.20 for satisfaction, with an average IOI-HA score of 24.17 [25]. Overall, participants obtained positive above average hearing aid outcomes measured through the IOI-HA.

**Table 2.** Hearing aid outcome results measured through the IOI-HA.

IOI-HA Item	Mean (SD)	Median (1–5)
Daily Use	3.91 *(0.64)*	4
Benefit	4.46 *(0.76)*	4
Residual activity limitation	4.75 *(0.59)*	5
Satisfaction	4.58 *(0.76)*	5
Residual participation restrictions	4.83 *(0.47)*	5
Impact on others	4.96 *(0.20)*	5
Quality of life	4.58 *(0.58)*	4
Total	32.08 (0.33)	32

**Figure 2.**
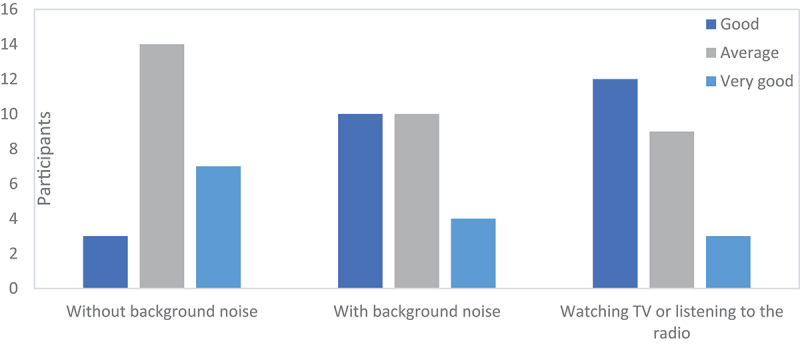
Hearing aid functioning in selected contexts (*n* = 24).

Regarding hearing aid functionality, all participants reported average, good, and very good performance in various contexts, including background noise, without background noise, and while watching TV or listening to the radio (Figure 2).

**Figure 3.**
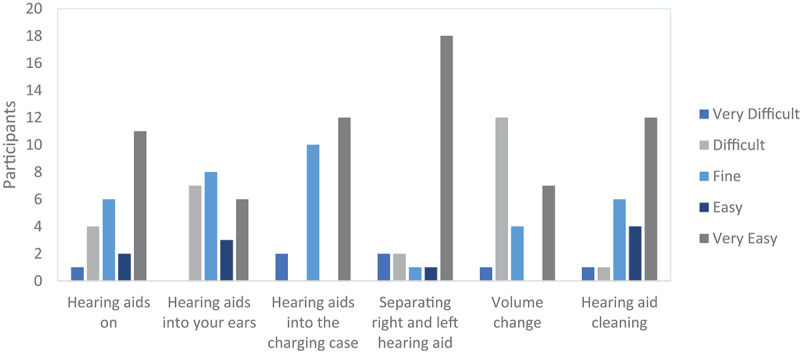
Hearing aid handling and care.

In terms of hearing aid handling and care, the majority of participants reported that it was easy or that they could perform handling tasks comfortably. These included putting the devices on, inserting them into the ears, adjusting the volume, and cleaning (Figure 3).

### Qualitative self-reported outcomes

In the thematic analysis of the open-ended qualitative questions, participants mentioned seven themes with hearing aid benefits, including improved hearing, communication, and social life mentioned on more than 20 occurrences (Table 3). Additionally, participants reported positive change in their communication, lack of stigma associated with their hearing aid use and positive impact of family on hearing aid use.

**Table 3.** Qualitative thematic analysis themes, number of occurrences and example quotes.

Theme (occurrences)	Example quotes
Improved hearing, communication and social interaction *(20)*	“She can hear better” “Yes, because they said to her now, they cannot gossip about her because she now hears”
Reduced dependency on others *(5)*	“No need for someone to help her explain everything, especially at the club”
Positive impact on social life and Family *(13)*	“She is happy now that she can hear better and communicate well with people” “The family is happy”
Gratitude and appreciation *(9)*	“It really helped me a lot because he can hear better and enjoy life happily and freely” “100% my family is happy”
Perception and recognition of Change *(9)*	“They see a big difference when she wears them; she hears better” “He does not see any changes to people now”
Positive treatment and acceptance *(13)*	“It changed my life very much because before I was avoiding conversation, now I can talk to people freely without fear” “They treat him good, with no problem”
Lack of stigma *(4)*	“No, because no one notices, and she is not ashamed of what people will say”

Participants reported that the mHealth support program was useful, with the following themes including (i) the useful role of WhatsApp for communication, (ii) learning and knowledge enhancement and (iii) assistance and support appearing in a high number of occurrences during inductive thematic analysis. Some strengths of the support program mentioned were that the messages received were clear and easy to understand. Suggestions for the mHealth program improvement included the incorporation of additional information and an increased use of IsiXhosa in the messages. Despite these recommendations, overall, participants expressed satisfaction and gratitude for the support program.

### Affordability and willingness to pay

Regarding affordability and cost, all participants indicated willingness to pay between US$2.6 to 10.5 (R50 to R200) for the hearing aids. The most frequently mentioned amount for the hearing aids was US$ 10.5 (R200). However, 16.7% of participants mentioned that they cannot afford to pay any amount for hearing aids due to income constraints and other financial commitments. In terms of income, all participants included in the project were receiving only an old-age social grant, which is less than 131.1 USD (R2500) per month.

## Discussion

The current study evaluated a CHW-led, community-based hearing aid fitting model within the Bowen feasibility framework. Several key insights were revealed. The high satisfaction levels among participants not only underscore the acceptability of the model but also reflect its responsiveness to user needs in low-income settings. The notable demand for the service is evident from participants finding their hearing aids extremely helpful and their willingness to recommend them, indicating a vital need in these communities. The successful implementation of the program, as planned, highlights its practicality and adaptability to resource-limited environments. Furthermore, the integration of a mHealth support program was instrumental in enhancing participants’ knowledge and self-confidence in handling hearing aids, thereby fostering greater independence and engagement with the technology. These findings not only align with Bowen’s framework but also contribute to a deeper understanding of implementing healthcare innovations in low-income settings, offering valuable insights for future policy and practice.

### Feasibility and acceptance of community-based service

Our study supports the findings of Frisby et al. [17], which demonstrated the acceptability, practicality, and feasibility of a similar CBR model within the same low-income community. The CBR service delivery model offered advantages, including flexibility and mobility of hearing healthcare services. While both studies employed CHWs, it is worth noting that Frisby et al. [17] utilized more expensive, advanced behind-the-ear, self-fitting OTC hearing aids (Lexie Lumen) as opposed to the preset-based OTC hearing aids employed in the current study. In comparison to the programmable hearing aids, the hearing devices used in this study are bud-style in-ear devices, more affordable (500 USD less), and require no programming before fitting. Despite being less advanced and more affordable, these devices yielded similar positive self-reported hearing aid outcomes compared to Frisby et al. [17]. Additionally, a feasibility study by Nieman et al. [16] on a community-based hearing care intervention (HEARS program) reported high acceptability among participants, with 93% reporting benefits and 100% recommending the intervention. Although Nieman et al. [16] and our study were facilitated by CHWs, it is important to note that Nieman et al. [16] provided PSAPs while our study used preset-based OTC hearing aids. The difference between the PSAPs used in Nieman et al. [16] and the hearing aids used in this study include that the PSAPs lack programming and other features like noise reduction or feedback cancellation.

The successful implementation of the CHW-based hearing healthcare provision model, as demonstrated in our study, illustrates the potential to replicate and scale hearing healthcare services in other low-income communities. Similar feasibility has been observed in other low-income communities, including India and Bangladesh. In India, a study conducted by Emerson et al. [26] demonstrated the effectiveness of trained CHWs in identifying disabling hearing loss and providing programmable mini behind-the-ear hearing aids. Consistent with our study, the outcome results reported by Emmerson et al. [26] measured through the Abbreviated Profile of Hearing Aid Benefit (APHAB) indicated improvements and benefits in communication during daily activities. In Bangladesh, a randomized control trial conducted by Borg et al. [27] revealed similar positive performance measured through the IOI-HA between participants fitted with pocket model hearing aids through a community-based approach compared to center-based approach. These studies support our findings, which revealed the feasibility and efficacy of CHWs facilitated hearing healthcare provision models in low-income communities.

### Hearing aid outcomes of community-based service

Our study revealed positive hearing aid outcomes measured through self-report questionnaires. For instance, the total IOI-HA score obtained was 30.2, with average scores of 3.7, 3.8, and 4.2 for hearing aid use, benefit, and satisfaction, respectively. On average, the results closely resemble those reported by Frisby et al. [18], wherein a total IOI-HA score of 32.1 was attained, accompanied by average scores of 4.4, 4.6, and 4.6 for hearing aid, benefit, and satisfaction, respectively. While the difference in total mean IOI-HA scores was minor (0.32), the variations in scores for hearing aid use, benefit, and satisfaction were significant. A potential contributing factor to the superior outcomes observed in Frisby et al. [18] could be the use of more advanced programmable self-fitting OTC hearing aids. Additionally, the performance of hearing aids in various settings, such as background noise, noise-free environments, and during activities like watching TV or listening to music was reported by Frisby et al. [18] to be above average. In another recent study by Nieman et al. [11], significant improvements in self-perceived communication function were observed in participants who received low-cost amplification devices (PSAPs) and instructions from CHWs compared to a waitlist control group.

### mHealth-supported CHW-Led hearing care

Some of the recommended best practices of community-delivered hearing healthcare were adopted during the implementation of this study. These included the use of already trained and experienced CHWs, the use of mHealth technologies, continuous education through the mHealth support program, and referrals to the onsite nurse for ear disease management. These recommendations are in line with a study bv Suen et al. [23] which first proposed some best practices to ensure success of community delivered hearing healthcare. These practices included competency-based training of CHWs, supervision by an audiologist or ENT surgeon, continuing education, a clearly defined scope for all members involved, adoption of existing policies on the provision of hearing healthcare, the use of technology, and the tracking of costs.

The mHealth support program significantly improved participants’ abilities to maintain and care for their hearing aids, with the majority finding these tasks easy to perform. These positive findings complement those from Frisby et al. [18], which demonstrated the feasibility and effectiveness of an mHealth support program in enhancing hearing aid acclimatization, as well as care and handling. While both studies report positive findings and were implemented in the form of WhatsApp and text messages, differences existed in the frequency and duration of the support program. Frisby et al. [18] employed a 45-day period with 20 messages sent whereas, in our study, fewer (14) messages were sent over a shorter (30-day) period.

### Scalability of community-based hearing care

The successful implementation of the CHW-based hearing aid model demonstrated in this study highlights the potential for replicating and scaling CHW-provided hearing healthcare services in low-income communities. While the feasibility of such models has been established, it is crucial to address their sustainability. This includes the ongoing promotion of innovative, affordable hearing products, such as GoPrime hearing aids. In addition to their affordability, these devices are also easy to maintain and use in LMICs as evidenced, for example, by their being rechargeable.

### Limitations

Despite the demonstrated feasibility in improving hearing aid outcomes, this study presents several limitations. The primary limitation was the relatively small sample size, comprising only 25 participants, and the consequent possibility of sampling bias. This study was purely observational, lacking control groups for comparison. The outcomes were not assessed over the long term, limiting our understanding of the sustained evaluation of the CHW interventions. The evaluation was purely reliant on self-reported hearing aid outcomes, excluding objective and behavioral measures such as aided speech in noise and in-ear measures. Finally, there were no matching pre- and post-outcome measures to evaluate individual differences in outcome measures post-hearing aid intervention.

## Conclusion

The community-based hearing aid fitting model, facilitated by CHWs in low-income communities was demonstrated to be feasible. Successful implementation may be due to several critical practices, including CHW training, the integration of mHealth technology, and audiologist supervision. While this model has proven feasible in improving hearing aid outcomes, it is important to conduct further research in a variety of other low-income settings to assess the scalability of the model. Comparative effectiveness studies should be conducted to evaluate its success in relation to more traditional hearing aid fitting models.

## APPENDICES Consent FormINVITATION TO RECEIVE HEARING AIDS

The hearX Foundation, in partnership with the hearX Group and the University of Pretoria, will be providing a hearing service in the communities. Many people suffer from hearing loss. This project aims to identify community members with hearing loss, demonstrate the potential benefit of hearing aids, and fit adults with suitable hearing loss with hearing aids.

### What will I need to do if I agree to receive the hearing aids?

One of the tests done at the previous hearing check will need **to** be confirmed before the hearing aids can be fit. This test will be non-invasive, without charge, and results will be made available to you. All testing should take about 30 minutes to complete. Should you agree, the following procedures will be followed:

- Questionnaire about your hearing (5 minutes)

Before you are fitted with hearing aids, you will be asked a few questions about your hearing and how you feel about hearing aids.

- Looking in your ear with a camera [Video-otoscopy] (5 minutes)

For this test, you will be required to be seated upright while your ear canal is visually inspected using an otoscope (ear-light).

- Hearing aid fitting (20 minutes)

The hearing aids will be set to a program suitable for your results. After that, you will be taught how to use and look after your hearing aids. You will receive phone calls on days 8, 20, and 43 to answer some questions. You will need to have a follow-up visit 4 weeks and 6 months after the hearing aid fitting, where you will be asked to complete two questionnaires. You will receive WhatsApp messages or SMSs during the 4 weeks to help you learn more about your hearing aids.

### Are there any risks or benefits for me if I participate in this study?

Participants will not be exposed to any risk or experience any discomfort during this hearing aid fitting. Participants can keep their hearing aids once the project has been completed. Information obtained from this study will assist in increasing the effectiveness of smartphone technologies in hearing loss detection, support, and intervention. Participants will also be referred to follow-up services when and where necessary.

### What are your rights as a participant?

Your participation in this project is entirely voluntary. You may decline to participate or stop at any time during the examination. This will not affect any current services you are receiving. You can consent if you wish to have your results used as part of any potential research based on this service. You can decline to have your results included in any potential research based on this service without affecting any current services you are receiving. You can provide your contact details should you be interested in participating in potential future research. You can decline participation in potential future research or request that your contact details be removed without affecting any current services you are receiving.

### COVID-19 Protocols

The hearing screeners will bring in hand sanitiser, and all participants, hearing screeners, and researchers will be required to sanitize their hands frequently. All equipment will be sanitized before and after each participant’s tests. If the participant, hearing screeners, or researcher feels unwell on the day scheduled for testing, the testing will be postponed to a later date.

### Confidentiality

All your information will be kept confidential. Once your results have been captured, a number will be allocated to your results. All data will be analyzed using the alphanumerical code assigned to you. Your name will not appear on any documents.

Should you consent to have your results used for research purposes, research articles in scientific journals will not include any information that could identify you. All the data collection sheets from this project will be stored for a period of 15 years in both hard copies and scanned electronic versions.

Before agreeing to participate, you should fully understand what is involved. Please do not hesitate to ask your hearing screener if you have any questions that this letter does not fully explain. Alternatively, you can contact us at info@hearxfoundation.org or send a please call me on 068 192 2413 (Mbekweni) or 084 393 0717 (Khayelitsha).

### CONSENT TO RECEIVE HEARING AIDS

I, _____________________________________________, hereby consent to:

**Table:**

I consent to receive the hearing aids.
I consent that my results be used anonymously for any possible research publications on this project.
I consent that my contact details be stored and that I can be contacted for any potential future services or research.
I consent that photos be taken during the hearing aid fitting and follow-up visits.

I have read or been explained the content of the consent letter verbally. I understood the consent letter and have been allowed to ask questions, and I am satisfied that they have been answered satisfactorily. I understand that I can keep the hearing aids once the project is complete. I know I may withdraw from the project should I wish to do so. I understand that every effort will be made to ensure that I am not harmed while receiving the hearing check.

**Signature**: _____________________________ **Date**: _____________________

**Phone number**: ______________________________________________

### Hearing Aid Serial numbers

Left: ______________________________________________________

Right: _____________________________________________________

Four week follow-up questionnaire

**Table:**

Participant number:
**Date:**
**Field worker number:**

**General comments**:

e.g., Were they wearing the hearing aid when you arrived, and what program were the hearing aids on?

______________________________________________________________________________________________________________________________________________________________________________________________________________________________________________________________________________

1. Please indicate your agreement with the following statement: ‘The hearing aids have improved my hearing’.

**Table:**

Strongly disagree	Disagree	Neither agree nor disagree	Agree	Strongly agree
❏	❏	❏	❏	❏

2. How well did the hearing aids work when you were having a conversation without background noise?

**Table:**

Very Poor	Poor	Average	Good	Very Good
❏	❏	❏	❏	❏

3. How well did the hearing aids work when you were having a conversation, and there was background noise?

**Table:**

Very Poor	Poor	Average	Good	Very Good
❏	❏	❏	❏	❏

4. How well did the hearing aids work when watching TV or listening to the radio?

**Table:**

Very Poor	Poor	Average	Good	Very Good
❏	❏	❏	❏	❏

5. Would you recommend these hearing aids to your friends and family if they had hearing loss?

**Table:**

Definitely not recommend	Not recommend	Neutral	Would recommend	Definitely recommend
❏	❏	❏	❏	❏

6. How easy or difficult is it to put these hearing aids on?

**Table:**

Very difficult	Difficult	Fine	Easy	Very easy
❏	❏	❏	❏	❏

7. Have you already replaced the domes of your hearing aid?

**Table:**

Yes
No

8. If you had to replace the dome, how easy was it to replace?

**Table:**

Very difficult	Difficult	Fine	Easy	Very easy
❏	❏	❏	❏	❏

9. How easy did you find putting the hearing aids into your ears?

**Table:**

Very difficult	Difficult	Fine	Easy	Very easy
❏	❏	❏	❏	❏

10. How easy did you find putting the hearing aids into the charging case correctly?

**Table:**

Very difficult	Difficult	Fine	Easy	Very easy
❏	❏	❏	❏	❏

11. How easy was it to see the difference between your right and left hearing aid?

**Table:**

Very difficult	Difficult	Fine	Easy	Very easy
❏	❏	❏	❏	❏

12. How did you find changing the volume of your hearing aids?

**Table:**

Very difficult	Difficult	Fine	Easy	Very easy
❏	❏	❏	❏	❏

13. How did you find changing the programs of your hearing aids?

**Table:**

Very difficult	Difficult	Fine	Easy	Very easy
❏	❏	❏	❏	❏

14. How comfortable was it to wear hearing aids in your ears?

**Table:**

Very uncomfortable	Uncomfortable	Fine	Comfortable	Very uncomfortable
❏	❏	❏	❏	❏

15. How helpful has the hearing aid support program you received on the phone been for you?

**Table:**

Not helpful	Help a little	Not sure	Helpful	Very helpful
❏	❏	❏	❏	❏

16. The information I received via WhatsApp/SMS after receiving my hearing aid was helpful.

**Table:**

Strongly disagree	Disagree	Neither agree nor disagree	Agree	Strongly agree
❏	❏	❏	❏	❏

17. Did you require assistance in performing any of the above-mentioned hearing aid tasks (10–17)?

**Table:**

Yes
No

18. If yes, which area did you require assistance?

**Table:**

Changing the volume
Changing the program
Charging the hearing aids or hearing case
Changing the domes
Putting the hearing aids in your ears
Cleaning the hearing aids.
Changing the wax guard

19. Do you have any concerns about wearing your hearing aids?

**Table:**

Yes
No

If yes above, can you please share what your concerns are?

______________________________________________________________________________________________________________________________________________________________________________________________________________________________________________________________________________

20. Did people treat you differently after you started wearing a hearing aid?

______________________________________________________________________________________________________________________________________________________________________________________________________________________________________________________________________________

21. Do you feel like WhatsApp/SMS information was sufficient to support you in using your hearing aids?

______________________________________________________________________________________________________________________________________________________________________________________________________________________________________________________________________________

22. What information do you think is lacking from the WhatsApp/SMS information?

______________________________________________________________________________________________________________________________________________________________________________________________________________________________________________________________________________

23. Did you ever go back and reread the messages or relisten to the voice notes to remind you how to use your hearing aid? If yes, which one?

______________________________________________________________________________________________________________________________________________________________________________________________________________________________________________________________________________

24. Was the information in WhatsApp/SMS easy to understand?

______________________________________________________________________________________________________________________________________________________________________________________________________________________________________________________________________________

25. Were there times during the day that you did not wear your hearing aid? Why?

______________________________________________________________________________________________________________________________________________________________________________________________________________________________________________________________________________

26. Do you have any questions about how to use or look after your hearing aid that the messages did not answer?

______________________________________________________________________________________________________________________________________________________________________________________________________________________________________________________________________________

27. Any recommendations to improve the support program?

______________________________________________________________________________________________________________________________________________________________________________________________________________________________________________________________________________

28. You can keep your hearing aids free of charge, but how much would you be willing to spend if you had to pay for a hearing aid?

______________________________________________________________________________________________________________________________________________________________________________________________________________________________________________________________________________

29. In what ways has using your hearing aids affected your life?

______________________________________________________________________________________________________________________________________________________________________________________________________________________________________________________________________________

30. Have your family, friends, or colleagues noticed a difference since you started wearing hearing aids?

______________________________________________________________________________________________________________________________________________________________________________________________________________________________________________________________________________

31. Were there any situations where your hearing aids did not help?

______________________________________________________________________________________________________________________________________________________________________________________________________________________________________________________________________________
